# The Present Needs in Dentistry

**Published:** 1895-05

**Authors:** J. Taft


### The Present Needs in Dentistry.

BY J. TAFT.

Read before the Mississippi Valley Dental Society, April 17th, 1895.

We may imagine one standing upon the threshold of the present century and looking with earnest inquiry into the future, desiring to learn, if he may, something of the development of dental science and art in the coming hundred years.

He looks about for data upon which to base opinions or draw conclusions, but, so far as dentistry is concerned, he finds little, if any, promise for the future. There is no soil in which it can grow, no environment from which any remarkable product could arise, it had attracted no considerable attention on the part of the public, nor even by those engaged in the healing art. The elements of progress were not visible, and here an inquirer, however penetrating his gaze or vivid his imagination, would have utterly failed to gain a glimpse of that which has been realized in the development and growth of dentistry from that to the present time. The common mode of forecasting the future is by a survey of the history of the past; but the adoption of this method for estimating the progress of dentistry would have been a failure.

Is it true that we of to-day stand as helpless to forecast the future as he of one hundred years ago? Perhaps not, for the growth of dental science and the discoveries in dental practice are before us; from these, we may legitimately draw some conclusion as to the future. The resources of the dental profession have grown to large proportions, especially when we take into account the brevity of its age.

The latter half of the century has witnessed the major part of the growth, notwithstanding this, we can not reckon with much definiteness of conclusion, as to what will be in the future, in the line of development and improvement.

Though the past growth may be but a shadowy index for the future, yet we may properly consider some points in the present state of our profession that may in the future be improved or changed.

I will invite your attention for a brief period to what may be regarded as some of the needs of the present time, and consider for a little, what may be done to answer these needs. And first, may be mentioned a lack of proper appreciation for dentistry as an honorable and useful calling. It is within the memory of man when the expression was frequently used, and even by men of culture, education and professional ability: "I am ashamed to be known in society, or even outside of my office as a dentist." Expressions of this sort are less frequent now than in the past, but language of this or similar import is heard even now; and in many cases where expression is not given to such thoughts the manner and bearing clearly indicate a want of just appreciation of the honor that should attach to those who devote their lives to the mitigation and prevention of ills and suffering to which humanity is subject. This is true not only of those outside of the dental fraternity, but, alas, of too many of those within it; and sometimes of those who are veterans in its ranks.

When we consider the great amount of empiricism and quackery that exists, and the difficulty experienced by the laity in discriminating between the truly professional dentist and the quack it is not a matter of wonder that both are placed, in popular estimation, upon about the same low level. An encouragement in this matter is found in the fact that many better prepared in general education and culture are now entering the ranks than in past years. Formerly it was the exception that a well educated person sought to enter the practice of dentistry; now a large proportion of those who come in are comparatively well educated and there is a constant improvement in this respect. This need is being supplied, but not sufficiently rapid to satisfy the desires of the more enthusiastic.

To our dental colleges, in the main, belongs the credit of effecting this change; most of them requiring a good English education and some even more than this. The want of a liberal education has been one of the impediments not only in the way of professional progress, but has also been an obstacle to the realization of that appreciation to which an honorable and useful profession is entitled. The educated dentist is better prepared to place a true estimate upon his profession than one ignorant and uncultured. He who practices his profession chiefly from a mercenary motive does not, either in action or words, honor it as he ought, nor is he capable of exercising the desired influence upon the public as he would under the stimulus of high and noble motives, viz; the intent and determination to render to every one who comes in his charge the highest possible service.

A large proportion of the students who enter our dental colleges have very crude ideas with regard to true professional status and honor. Many enter upon the preparatory training with no higher ideas of the character of his prospective calling than the apprentice to the blacksmith or carpenter. His mind too often is upon the question, how can I make the most money, and how can I most quickly gain notoriety? Now, the correction of all these false conceptions devolves in the main upon those who have in charge the student's professional education and training. This is quite as important as his instruction in the principles and practice of his profession, but it may be said as it often is; "It is not the business of the dental colleges to teach ethics." In this day, however, there are few indeed who will clearly and pointedly venture this criticism. Why should not he who is helping the student in preparation for his life work, do all he can to make that preparation as complete as possible; not only store his mind with the knowledge of principles and train his fingers to the highest manipulative skill, but also give him such ethical training as will best prepare him for a successful career; often this is as much dependent upon deportment and correct manner as upon knowledge and skill. I do not hesitate to affirm that no teacher does his full duty to those in his charge, who neglects the inculcation and fixing of true ethical principles, not only in the mind, but stamping them in to the very core of his being. Progress is being made in this direction, but there is room, and indeed great need for improvement yet; but it is coming.

At this point, permit a suggestion, or rather an inquiry in regard to equipment and facilities for giving instruction in our dental colleges. In this matter I am free to say that upon the whole, never were the dental colleges so well prepared for the accomplishment of their work as to-day. I have naught but commendation for a large proportion of the faculties of our colleges. It may with fairness be said that they are earnest and faithful in their work, and as successful as can be expected with their facilities and enviroments. In regard to the curriculum of some of our schools, at least, there might with a decided benefit be an extension; branches ought to be included that are omitted altogether. This, it may be observed, is being remedied. Additions are being made each year. Extention of the curriculum usually requires additional teaching force. The condition of buildings in which much of our college work is done, is so defective as to render it impossible to accomplish the best results. Quite a number of the colleges of our country have secured model buildings, others are hoping in the near future to secure adequate accommodations; but for the present there is great want in this respect.

In the present important methods of teaching, ample room and proper arrangement are required for the accomodation of each department of the work. The best and most approved equipment should be employed. Good light is a necessity. Natural light is always best. Artificial light should be avoided whenever possible. Thorough ventilation should always be secured. The arrangement and environment ought to be pleasant and inviting. These conditions should receive the earnest attention of every educator in the profession. Every dental school should have a museum fully furnished and supplied with every preparation serviceable for illustration in teaching; preparations of the natural organs, as well as enlarged models of the same are indispensable for the full presentation of many subjects. Knowledge is communicated through the eye as well as by the spoken language to the ear, and indeed many receive knowledge more readily and more effectively by means of sight than by the spoken or printed word—hence, the importance and value of facilities for object teaching. Quite a number of colleges have more or less material proper for a museum, but none have all they need, and it may well be questioned if any have the material now in their possession arranged systematically as it should be to serve the best purpose. What would be thought of a literary or scientific institution of learning without a library or museum? The general verdict would be, deficient in equipment, therefore defective in work.

In addition to a good museum, there should be in every dental college a good library which should embrace all the standard works on the science and art of dentistry and a complete series of the dental journals, especially those in the English, and if possible, those in foreign languages, also the published transactions, so far as attainable, of all dental societies should have a place in such a library. In addition to these, every work that has been written in any language, in the past, so far as possible, should be included. In such a library other books than those strictly pertaining to dentistry will often be found desirable, such as dictionaries, works on comparative anatomy, hygiene, etc., etc. A well arranged and systematized library will be highly appreciated by earnest and educated students. The manner of utilizing such a library will be modified and determined by the number of students desiring its privileges. It would not be practicable to make such an one a circulating library, and hence, a well arranged and orderly reading room in connection with the library is a necessity. A few of our schools have done something in the way of providing these facilities and are doing what they can for increasing them. None that we are aware of have a completed equipment in this respect. In some of our schools at least, there is an entire want of interest in this matter, but attention is being more and more turned to the supplying of this great need.

The same criticism in regard to dental teaching may be made, that is often indulged in, in regard to the course usually pursued in the teaching of general medicine, viz: that there is relatively, at least, a deficiency in the study of hygiene—the laws of life and health: The chief effort is expended in the consideration of disease, its phases and management, and altogether too little attention devoted to its prevention. While the highest skill in treatment of diseases should be exercised, it is not as important as the ability to ward off and prevent recurrence of disease.

Doubtless there is room for improvement in the text books used in the dental colleges; up to within recent years these for the most part, have been prepared with reference to the general practitioner more than to the requirements of the student while the latter ought in some sense, to have the first consideration; and more now than ever before, for though the college is the only recognized way of entering the profession, and a far larger number are coming in, than ever before, at least sufficient in number to entitle them to some consideration, in the matter of proper text-books. The practitioner relies more upon the periodical literature for his reading matter. than upon the standard works, and it is by no means so important now to have such works as in former times; but it is more important, to have properly prepared text books for the student than ever before.

An effort was made a few years ago by the National Association of Dental Faculties to have this want supplied, but the effort has not been as successful as was anticipated. A few responces have been made to this effort, but a great need still remains to be met in this direction. When this is accomplished another much to be desired change will be put upon the highway to hopeful realization, viz.: harmony, and unification in modes of teaching, and in requirements. It is gratifying to know however, that influences are in operation leading up to this desired object, and mainly through the National Association of Dental Faculties." This organization has done more for the promotion of harmony, unity, concert of action, and procedure, than all other agencies combined. Through the instrumentality of this body, the standard of entrance requirements has been much advanced, the curriculum and coarse of study made broader, the requirements for graduation have been raised, the qualifications, and endowment of prospective students, are now more closely scrutinized than ever before; and the time of the annual session has been, and is being increased. Justice requires this statement, that all this has not been wholly accomplished by the Association above mentioned, but that it has been the chief factor, none will deny. The American Dental Association has for years devoted its influence to the advancement of dental education, giving encouragement and counsel, to those aiming for the best things, and uttering caution to those who persistently go on low levels, or indulge in ways adverse to the interest to a true professional status.

The National Association of Dental Examining Boards, has exercised a wholesome influence upon the dental colleges of the country.

Now in view of all this, let us not conclude that Utopia has been reached, that nothing remains to be done. In nearly all the particulars of progress above mentioned, let us bear in mind, that only a transition state has been reached, that and the responsibility of carrying onward and upward, the work so well begun rest with the men of to-day, such as those who constitute this Pioneer Body.

When we look abroad over our professional field, and find so many needs, so many lapses, so many voices out of tune, so many movements in the wrong direction, it is no wonder that discouragement takes hold upon us, but let us not yield to its depressing influence, there are also many things to encourage. Indeed we can look with pleasure on many things pertaining to our profession, and perhaps none for which we should be more thankful than for the unity and solidity that are prevailing characteristics in its ranks. There is no element of discord to produce contending factions. Occasional agitations that have occurred have after a brief period passed away. Our profession presses forward in solid column to the accomplishment of the great objects of its being; and while all its members are supposed to be independent thinkers, and capable of drawing their own conclusion, and deciding mooted questions, yet whenever important matters affecting the whole body arise, there is great unanimity and harmony of views and action.

Nowhere can a class of men be found, so free, yet so firmly banded together, without division or factions. The frequent coming together of the profession in more than a hundred dental societies in all parts of this broad land, for a common purpose, with a common sympathy and strong fraternal feelings, emphasize in a marked manner the unity of the dental profession; thus do we occupy enviable ground, which should be utilized in discharging the duties and responsibilities devolving upon us. We have no intestine struggles in which to spend our strength or divert our energies and naught to do but go on and fill the high behests before us. Is it then a marvel that such great and rapid progress should be made, and that in the race for high achievements dentistry should be abreast of even the foremost? Let us be careful that our work does not lag. The dental profession is one of the battalions of the great army engaged in the warfare against disease and the ruin it works. Let us then press on to that great victory, when disease shall be conquered and death shall no longer sit regnant as "King of Terrors," but shall be relegated to the position of the kindly porter to open the gate for the easy passage of redeemed humanity, from this to a higher sphere of activity and enjoyment.

DISCUSSION.

Two of the gentlemen named on the programme, to discuss the same, Doctors Crouse and Bethel, being absent Dr. Sage, of Cincinnati, the remaining discusser, spoke as follows:

Mr. President and Members of the Society: I am constrained this morning, in rising to address this Association, to do what I have often deprecated in others, that is, apologize for lack of adequate preparation. Time for such preparation has been short and my opportunity limited, and I do not feel myself in a position to discuss the subject with the thoroughness which its importance merits. In listening to the reading of the address by our President; however, there were several points to which I am particularly constrained to give attention and to touch upon briefly. The first is, with regard to the statement which Dr. Taft makes in his opening, in substance that the dentists, as a body, are not sufficiently self-respecting; and that the dentists as a body are not, therefore, held in the high esteem to which we feel our profession entitled from those outside, meaning, I presume, our patrons, those who come to us for our services. This is a singular fact, which from my first entrance upon the practice of dentistry I have observed. And, if I mistake not, at the very first convention which I ever attended, and which, by the way was a meeting of this same Mississippi Valley Dental Association, this very matter was brought up. I then thought and still think, that dentists are not sufficiently self-respecting, and are not held in the high esteem that those in other professions are regarded. Now, accepting this as a fact, we come to the inquiry, what are the reasons of it? Why are dentists not sufficiently self-respecting let us first ask. In other words, why do they not thenselves hold their profession in sufficiently high esteem? I admit that there are in the profession many men who cannot be charged with a failure to properly esteem their profession, but who give themselves heart and soul to its practice, and have dedicated their best efforts to its advancement, and never for an instant deviate from their devotion to it. Such men are never distracted by the allurements of other professions, but remain content to be dentists and dentists only. For my part I have always envied men of that particular description; while I have sometimes, I will confess, stopped and asked myself the question—and if I say anything heretical here I am ready and willing to be called to order, and receive any admonitions in the way of keeping my mouth closed that any member chooses to give me—but I say the time has been when I myself have stopped to consider whether, in view of the laborious effort put forth and required of the student of dentistry in preparation for practice, and considering all the further pains-taking effort necessary after he gets into practice, whether the average student would not do better in some other calling? That question, I am afraid I must confess, has arisen in my mind more than once. I have thought, with respect to the profession of the law, whether I could not, had I followed that which is generally recognized as the leading profession, and the one which furnishes the highest rewards and the most tempting perquisites of all the professions,—I have somtimes wondered whether I could not have made more money and have acquired more influence, and exercised my natural talents better in that sphere than in the one I chose? I say, I have sometimes asked myself that question, in the past; but now, so far as I personally am concerned, I am satisfied to answer that question in the negative. I think not. I think I know what the qualifications of the lawyer are, and what a lawyer must be in order to achieve success; and I do not think, so far as I am concerned, if you will pardon the egotism, that I have the qualifications which would have enabled me to become eminent in the practice of the law. Then the other professions, of medicine and the ministry, generally recognized as the leading professions—for, however it may be considered as derogatory to our claims in the dental profession, according to the strict definition of the term "profession" in "Webster", we are not in the category as professional men. We are rather what may be designated as semi-professional, not strictly professional, according to the definition, which describes a profession as a body of men engaged in any pursuit, not mechanical, agricultural or the like,—which definition, you will see, does not fit us altogether. So far as I personally am concerned I have been content to let these matters go. I don't care whether ours is a profession, or not; although I think we have a perfectly good right to claim to be a profession. The dental body, however, in the ordinary acceptation, does stand in an anomalous position in relation to the other professions; it occupies a position almost impossible to define; the metes and bounds of its proper functions have not as yet been ascertained and established. We can extend them almost indefinitely, as for, example in the direction of the treatment of tumors of the mouth and oral surgery, and to other departments now pursued by other specialists, if we choose. From my own stand-point, I consider, however that our proper province is to improve our present methods, not seeking to enlarge the boundaries of our ministrations. The public do not expect of us that we be physicians. I

do not mean by this to say that no one who practices either profession may not practice both, if qualified. But the public will measure us, so long as we profess to be dentists and invite them to come to us for ministrations in that direction, according to our abilities as dentists. While then every thing should be done to elevate and dignify our profession, it should be done in the direction of elevating dentistry in itself; not that the other may not be superadded, but let us stand upon the dignity of our own endeavors for the alleviation of human suffering in the lines we have long established, and not attempt to cloak our imperfections, if such may be justly imputed to us, by any borrowed splendor such as may come to us through a forced alliance with medicine or surgery. Dentistry will be best dignified by perfecting its methods, and so deserving the esteem and the gratitude of the public whom we serve and whose regard we would like to have. I say, then, that the discontent which some men feel with their own profession of dentistry, grows out of mental comparisons of their own standing as compared with that of other men in other callings; yet this is all comparative, of course, and I don't see anything in it to my mind of great importance. You will find in the villages and smaller towns lawyers who are supposed to be men of learning and high mental acquirements, who are not respected merely because they are lawyers; perhaps they are without briefs or cases, seldom appear in court, unless it might be, to champion a small suit in a squire's court, nevertheless, they have a certain following and command respect as men, though not as lawyers. On the other hand, the dentist around the corner, in the same village, having his office over some little grocery, perhaps, is constantly busy, is making money, and is esteemed even more highly than the lawyer in that community, even more highly than the physician, if he has a larger practice. Therefore it appears, that looking at the matter from the stand-point of the public, it is a question of money that measures the respectability of the dentist; if the dentist's wife wears a seal-skin cloak he will soon command the respect of the community. This, however, has nothing to do with the inherent respectability of dentistry; it is a respectable calling of which none of us ought to be ashamed; we should rather be proud of our profession. It all resolves itself down to the question of individual qualification. If a dentist is a gentleman of refinement, and of intellectual attainments, and adds to these qualifications, industry and social standing—and we will include, money—for that is indispensable in some communities—I say he will be respectable, and his calling will be respected. I used sometimes to think, when I first came into the profession, that there was too much of a desire to elevate dentistry by adventitious means, that there was too much of an attempt to put it on a factitious and fictitious basis. Attempts were being made to raise it a little above the grade of a trade, in which latter light the public seemed disposed to regard it. And for this view of the matter the public could not be altogether blamed, since it is but a half-century since dentists were held to be the same as barbers, and in fact sprang from the ranks of the barbers. Time will make all this right. In this half century, dentistry has made magnificent strides. The public are hardly to be blamed if they can not yet fully realize the advancement made. But they are learning, and when you meet a man in the street now he calls you "Mister." Perhaps you have a new servant-girl, and she calls you "Mister;" and people come to the door, and say, "Is *Mister* Jones in? I want a tooth pulled." That is the only idea many have, even to this day, as all had once, that the dentist is a tooth-puller! They don't know anything about the treatment of teeth—don't care anything about it, don't appreciate it, unless you do it without any pain; then they do appreciate it sometimes. But these things will all change in time; all we have to do is to attend to our business, perform our work in the world properly, thoroughly and conscientiously, labor as best we can to alleviate the sufferings of man and woman-kind, put their teeth in order, and avoid quackery, and we will reap the desired reward in time. It is not necessary for us to do anything further about it, or resort to any outside influences to bring it about; but, at the same time, I don't fail to appreciate the instruments, the means that are being employed to elevate dentistry, adventitious outside means, if you so choose to term them. This meeting of this Association is one of them. What do we meet here for? Not merely to exchange ideas of practice of dentistry, not merely to consult with one another as to how to fill roots; we know that although a secondary and remote object it is none the less an important one, that we impress the great public. We all know that; we don't say much about it, but that is true; and it is right that we should aim to do that by all proper means. It is all right. And we must impress the public with the idea that we are better than mere tinkers, or they will never be got to believe it. People are not grateful to their physicians; they are grateful to the trained nurse that comes to the bedside, and under the instruction of the physician, mark you, attends to their wants and alleviates their sufferings, prepares the medicines, and administers to their needs. They are grateful to the nurse, but not always to the physician, who is further back behind the throne and whose skill and pains-taking research has made all this possible, and has brought about their cure. They don't think so much of the physician. And so it is with regard to their estimate of us; they come in, and if we treat their teeth, stop their tooth-ache, they go off and don't think of us again until the tooth begins to hurt again, or the face swells up; then they come back, not because they are grateful, but because they have the tooth-ache again. They will learn to be grateful as time goes on. They will find out—it is astonishing how many do find out—that we are not mere tinkers.

Another point that Dr. Taft made I was interested in, was what he said with respect to the difficulty which the public have to discriminate between the quack and the professional dentist. If I had not known the Doctor so well I would have thought he meant to be amusiug and satirical there. That is one thing we have to contend with, the opposition of quacks. They use the same instrumentalities, and can fit up their offices as well as we can; they can administer to their patients as well as we a good deal of the time, and it is a hard thing for us to make patients believe that they are quacks. I know I have had patients come into my office and say "I went to some Steam Dental establishment, and they can pull a tooth as well as you can do it, and I believe a little better (laughter) I went there and got a bridge put on. You would not put it on; you did not think it a proper case; and I have had it on six weeks, and I am satisfied it will last sixty-five years." That is what we have to contend with. It is useless for us to say to them that we have been practising twenty or twenty-five years, and this other somebody has been practising but four or five years, and cannot know as much as we do. We cannot tell them that it is not in accordance with the code of ethics. We cannot do that in our business. But still, I think those things will right themselves in time.

Again as to the matter of elevating the profession, although I am not much concerned about it; you must get men to quit cutting prices in the small towns; every one wants to make a plate for less than his neighbor this can only result in bringing the estimation of the work to the level of the tinker's and cobbler's. That does not tend to increase the respect of the public for us. Not that charging big fees is to make us respectable; I cannot do that all the time; but I don't regard myself any the less respectable on that account.

We have too, to avail ourselves of these outside aids, such as the study of medicine. Even though we don't use the knowledge of medicine the public will set more store by us if we have taken such a course. I think the time will come when all will have a medical degree; I hope so. Or, at all events I am willing to pay this compliment to the dental colleges, that I believe with their extended course of study, they will prepare their students as thoroughly as the medical colleges. I don't know whether in the future there will ever be any final fusion; but dentistry is practically a specialty of medicine, although all dentists are not medical specialists, unless they have a medical degree.

Another point: Nobody, I presume, has ever questioned the propriety of instructing dental students in ethics; at the same time the students in our dental colleges, who come and pay their fees for instruction in dentistry ought to be taught all they can acquire in the college pertaining to the practice of their profession and professional ethics. I have sometimes also wondered why they have not established in dental colleges a chair of *belle lettres*, such as they have in classical colleges. Then the students need to be enlightened as to the how to do it and how not to do, in their occupation as dentists. Let them be instructed as to the management of the details of the business part of their profession, such as the amount and collection of fees, the reception of patients, etc. We can all look back over our earlier experiences and see where we could have avoided this or that error if some one had apprised us in advance of the little things that it is necessary to know, and which taken together make up the sum of business life. I do not consider such instruction beneath the dignity of a Dental College. It should embrace in its curriculum everything of practical value pertaining to the profession of dentistry.

I don't know that I have anything more to say on this subject and will make room for those who may have other points they wish to speak upon. There are others which deserve a thorough discussion.

On motion of Dr. J. TAFT, duly seconded, the privilege of the floor was extended to all present, whether members or otherwise and all were invited to participate in the discussions.

DR. HARRY B. RESPINGER, of Genava, Switzerland, was called upon and responded as follows:

MR. PRESIDENT, Ladies and Gentlemen: I will say a few words with regard to the practice of dentistry in connection with medicine. There has been, as I notice in the dental papers a great deal written in regard to this matter. In Europe, as well as in this country, we are tending to an advanced education of dentists, the giving of a good foundation, but I believe we may be going too for. I think that it is a mistake to require of a dentist a thorough medical education. While it may be well enough for him to have such knowledge, yet I do not conceive it to be any more a necessity than it would be for a pharmacist to have a complete medical degree; or pursue a course of study in medicine proper. I think it well to lay a sound foundation, but I think in many instances we may be going too far. I believe that by such a course we don't really advance the practice of dentistry. I do not think that a young man should be required, as is done in

Europe, to spend three years, or four years in studying medicine proper, and then to give to dentistry itself only a year or a year and a half. I think that is entirely a mistaken idea. Dentists in former days had medical knowledge, certainly, perhaps, very inferior to what we have, but they spent the most of their time in preparing themselves for their profession, by which they were to do good to mankind, and they were often men fully as capable as those of the present day. And thus, if I had to give my opinion on the matter, I should say, that I believe the curriculum of study as required in this country is certainly as good if not superior to our curriculum in Europe, where we demand of our dental students a two year's medical examination and a quantity of other matters which really afterwards are thrown aside. Not that I will say that it is useless, but it does not advance the student in his profession for which he is preparing. There is, however, one thing which I think is more important than the gaining of a professional education, that is, of a professional medical education ; and that is, to require a general liberal education. I believe by this means dentistry can be much further advanced than by the medical requirements. I do not certainly know it, but I believe, that in many schools in this country young men are permitted to enter who are really not far enough advanced in general education, they have not attained the proper standard, their intellect is not prepared for it, and thus their prescribed course of study is fruitless. I believe there should be a standard of education required, and that no young man should be permitted to enter a dental college until he has given proof, either by examination or by diplomas, from high schools that he is fully qualified to enter the dental profession. Thus, if I were to resume what I have said here, it will be, that I believe, that the profession at large could be further advanced by demanding from the students a liberal English education than by requiring students of dentistry to sit together for years on the same benches with medical students, and take a lot of degrees in subjects to which they cannot pay the same attention or take the same interest in, because they know that afterwards they will not be of immediate use. And by an English education I do not mean the preparatory education such as is demanded in England or on the Continent of Europe, where they require of dental students six or eight years of Latin and as many years of Greek. I believe that is perfectly superfluous. Certainly, a Latin education, such as can be obtained in three or four years is, to my mind, perfectly sufficient, perhaps even more than enough. Such an education is very good for understanding difficult expressions which may be used at will. I really think an education necessary, sufficient to qualify a man, after he has entered his profession to write a paper without making forty or fifty mistakes in five or six pages, such as I have seen them do here in our Universities. Students should have a full knowledge of the English language, and should be required to know at least one more (Foreign) language, so as to be able to follow papers, in the different periodicals, which may be written for for the profession at large. I believe that is all I have to say on the matter. It is of no great importance; but it is my idea, and I have been pleased to state it.

Dr. H. A. Smith: I have nothing especially to say. I have listened to the address of President Taft, and it occurred to me that if he would read that to the Faculties Association it would be well. Dr. Sage, in his somewhat protracted speech,—(Dr. Sage: I asked you to call me down)—said he was out of wind. Dr. Sage gave us some sage advise on business matters; but he is wrong when he intimates that the Dental Colleges do not teach their students ethics, and that they do not discuss fees in a mild way, also. The people who get the best fees never say much about them anywhere, even to the patients. What I wanted to say to you was that Dental Colleges do teach ethics; they teach their students, impress upon them as much as posible the better methods in business, and in conducting their practice; therefore, it is not necessary for us to take another course in the dental colleges, but go around and visit the institutions and become better informed than to get off such statements as those.

Vice-President Ames then called upon Dr. Harlan, of Chicago, who spoke as follows:

Mr. President, Ladies and Gentlemen: I listened to the President's address with considerable interest, and to the address of Dr. Sage. I do not know that I care to criticise the President's address, because most of his suggestions and most of his desires for the present needs of dentistry meet my own. In discussing some of the points that were brought up by Dr. Sage I should like to dispose of the quackery question first. The number of dental quacks, or the number of quack establishments in any city of any size do not interfere with the legitimate practice of dentistry to any extent anywhere. I think in the city of Chicago, where there may be thirty or forty advertising establishments of that kind, that legitimate dentistry does not suffer, but holds its head triumphantly above all such methods of gaining patronage; and certainly holds itself far above the operations that are performed in such institutions. Legitimate dentists, are largely, men of judgement and education and skill; and if they combine these three qualifications, why, there is no quack establishment in the world that can stand by the side of them. Legitimate dentistry is practiced honestly; there is no attempt to deceive. That puts every legitimate physician or dentist on a footing upon which any legitimate practitioner can stand.

With reference to the thorough education of dentists, it is almost universally put into practice; the dental degree in the United States is essentially a D.D.S. degree; the dental degree in England is L.D.S., or Licentiate of Dental Surgery; and the dental degree in every country where there is a dental degree is separate from the medical degree; there are only two countries in the world that require that dentists should have a medical degree, and those are Italy and Austro-Hungary. In Russia and Finland and Germany and other countries there is a separate dental degree. It is universal. The degree may not always be designated by the same name, but the requirements are about the same; in other words they all have a dental degree that is applicable to dentists and to dentists only.

The very excellent remarks of the gentleman from Switzerland, with reference to the education of dentists I heartily approve; that is, if a man has to spend a certain number of years, it is much better that he should spend some of those years in the acquirement of a foreign language than in being grounded in some department of science, or in some branch of technical knowledge which will prove useless to him in his future practice of dentistry. The curriculum, in the fundamentals, it seems to me, should be—and practically is—the same; but when you come to the point where you start off into the theory and practice of the art and science of medicine, or the art and science of dental surgery, then the paths diverege, and the education of each in his particular line should be distinct from that of the other. Prior to that there should be no distinction; and in the minds of the best educated people in the world is not any distinction.

Now, as to the estimation in which dentists are held by the public, that is largely a matter that depends upon themselves. If dentists are simply money-grubbers, as some physicians are, the public estimates them at their own worth. If a dentist is not progressive, why, he stands in the same relation to the public that the unprogressive physician does, or the lawyer. In every community that has been long settled in this country you will find that the dentists are aldermen and common councilmen, and members of boards of education, mayors and county officers, etc., the same as their fellow-citizens, where such political duties will not interfere with the proper exercise of their profession. In Illinois we have quite a number of men who are members of the legislature; but dentists as a rule do not seek political preferment of that kind, who are engaged in dentistry or medicine. It interferes with the consecutive attention to their practice, and in that respect is many times detrimental to them. There is no reason why men should not serve on the board of education in the city of Chicago or Cincinnati, or that a dentist should not be a member of the library board; or that he should not be a member of some other board of that kind. I think that only twice in the quarter of a century that I have lived in Chicago, have I known a dentist to be a member of the board of education; but I think this state of affairs is due more to their evident lack of desire to give the necessary time to the performance of such duties, than because the mayors during that time have been unwilling to appoint them. I know two or three instances where members of my profession have declined honors of that kind, because it would take so much of their time.

The working years of a dentist, are shorter than those of a physician, on account, principally, of the physical labor that a dentist has to perform, which many physicians do not; and so, when a dentist arrives at sixty or sixty-five years of age, many times, he is unable to endure the physical strain, which a physician is not called upon to undergo; and the dentist's advice at that age, perhaps would not be sought for, when that of a physician of the same age, would be. So we can not parallel the two professions from the beginning to the end of their career.

I believe that the dental profession is growing in the estimation of the public, and that, in spite of what Dr. Sage says, there are very many grateful people—grateful in many respects.

The question of fees, I think, might with propriety be discussed occasionally; but what my fee should be for a certain operation might not be the one that you would require or desire, and so you can not establish any absolute uniformity in that regard. I do not think there is any minimum, or maximum, that you can establish, that will meet all cases, unless it should be outrageous in either extreme.

The teaching of ethics in dental colleges is a matter that I thought had received attention. It perhaps receives more attention now than it did twenty years ago, or before that time. I should say that ten lectures during a term might well be devoted to a combination of ethics and jurisprudence; and that it would not be a bad idea for any and every dental college to establish a lectureship or professorship, that would embrace those two subjects; and they might include in the ten lectures one or two devoted to a consideration of the requirements of dental practice.

While I am on the floor I would like to say that this is the twenty-third year since I became a member of this Association; and I am glad to see that there is such a large number present to-day.

Dr. Sage: In what I said about the practice, I wished merely to account for the estimation in which dentists are held, generally. In many cases the quacks perform operations which are for the time being satisfactory, and apparently as good as those of the honorable dentist, notwithstanding that deception is practiced by the quack and he will in the end be found out. But the public, in the present apparent success do not always discriminate; they do not consider the eventual injury; and this is what is holding dentistry back. I say that the fact that there are such quacks, as Dr. Taft says, operating in the field of dentistry, is calculated to confuse the public as to who are honorable practioners and who are not. That drawback to regular and honorable dentistry will be overcome in time.

President Taft: The Executive Committee has not fixed the hours for these meetings; has anyone a motion looking to that?

On motion of Dr. Betty the hours of meeting of the sessions of the convention were fixed at 9 to 12 m. and 2 to 5 p. m.

Chairman Ames: What is the pleasure of the Association in regard to the present discussion? Will we continue it in the afternoon session?

Dr. Taft: There might be a half hour devoted to the continuance of this discussion. There may be other members desiring to speak, and we would like to hear from them. I offer that merely as a suggestion. Our time, of course, is limited.

On motion it was resolved to devote the first thirty minutes of the afternoon session to further discussion of the present topic, each speaker to be confined to five minutes.

On motion adjourned until 2 p. m.

AFTERNOON SESSION.

Convention met pursuant to adjournment, Dr. J. Taft in the Chair. The Chair requested those desiring to participate in further discussion of the topic last under consideration to respond promptly, as it was fifteen minutes past the hour set for reassembling, and reminded speakers of the five minute rule.

Dr. W. H. Gillette, of Cincinnati: Having been present this morning, I was very much pleased and gratified at the address of our President. I would not deign to say very much on that or mar its beautiful contents; but some of the remarks made as an outgrowth upon that, I beg to differ with. It was stated that the quack is not a detriment to the profession generally. With all due respect to our Chicago brother, I beg to differ with him. It might not hurt some members of the profession, who are away up on top; but quackery does hurt those who can least afford to be hurt. I speak from personal experience. And if those of us who are in the lower ranks of the profession are affected by this thing, those in the higher positions will ultimately feel its effects. If the base of the mountain is not fixed its top will eventually tremble and fall. Probably the gentlemen who have preceded me have been on the top, and never started from the base; they are honest in their opinions, no doubt. But there are thousands like myself who start out each year, and who have to battle with the elements of the world, and who have not any amount of money to begin with, and we have to cope with the close competition, and one thing and another, of the cheap dental establishments, cheap advertising concerns and cheap private concerns, and the dental colleges. I must say that though I admire and love knowledge, wisdom and intelligence; yet they alone will not support men. A man may have all the wisdom, all the understanding, that is desirable and necessary to run a dental office, but what does it avail him, if he has not the dental office to run? Now, I mean by this, that I have seen men possessing great ability, great understanding, great intelligence, and getting a large fee, whose work was not worth absolutely anything. So that knowledge, wisdom, understanding, much as they are to be desired, are not all that is necessary in order to be successful dentists. Knowledge is power. Knowledge is power, and it is not. It is powerful in so far as we can apply it; but if I possess all the knowledge in the world, and have not the opportunities to apply it, then it goes for nothing. So I say that quackery does hurt. Just below me there has started out, as I see by the sign, so-and-so dentist; he has matriculated in one of our colleges, but he has not a certificate, has not passed the State board, or anything of that kind. Of course we have a way to govern that; we simply can shut him out; but does that right the wrong? No. After we shut him out what we want to do, I think, is to make the requirements more strict. The dental colleges may have the requirements, but are they on paper, or do they require certain things that ought to be, from those who are going to enter into the colleges, and expect to graduate therefrom. Now, knowing this, are such men, are such quacks eligible to go through the dental college? I think something ought to be done to protect the profession.

Dr. Taft: The half-hour is not yet consumed; has any gentleman anything further to offer? If not we will pass to the next order of business.

Dr. Fletcher, of Cincinnati: It seems to me that the author of the paper should have an opportunity to close the discusion before it is passed, if he has something further to say. I would be glad to hear from our President, if he has.

Dr. Taft: I have nothing to say on the question which is not stated in the paper itself. There is nothing I care to discuss. The time is about up, and as our program is very full we will be compelled to work very closely to it, if the ground is to be all covered between this and tomorrow evening. If there are any further remarks we will hear them; if not we will proceed to the next subject, which is a paper by Dr. Callahan, on "English Tube Crowns for Bridge Work." Is Dr. Callahan present? (No response.) Dr. Callahan not being present we will proceed to the next subject.

[*To be Continued.*]

